# Prognostic value of HLA class I, HLA-E, HLA-G and Tregs in rectal cancer: a retrospective cohort study

**DOI:** 10.1186/1471-2407-14-486

**Published:** 2014-07-05

**Authors:** Marlies S Reimers, Charla C Engels, Hein Putter, Hans Morreau, Gerrit Jan Liefers, Cornelis JH van de Velde, Peter JK Kuppen

**Affiliations:** 1 Department of Surgery, Leiden University Medical Center, Leiden, The Netherlands; 2 Department of Medical Statistics, Leiden University Medical Center, Leiden, The Netherlands; 3 Department of Pathology, Leiden University Medical Center, Leiden, The Netherlands

**Keywords:** Rectum, Rectal cancer, Immune surveillance, HLA Class I expression, Foxp3+ regulatory T cells, HLA-E, HLA-G, Outcome

## Abstract

**Background:**

Evasion of immune surveillance and suppression of the immune system are important hallmarks of tumorigenesis. The goal of this study was to establish distinct patterns that reflect a rectal tumors’ immune-phenotype and to determine their relation to patient outcome.

**Methods:**

The study population consisted of 495 Stage I-IV non-preoperatively treated rectal cancer patients of which a tissue micro array (TMA) was available. Sections of this TMA were immunohistochemically stained and quantified for presence of Foxp3+ cells (Tregs) and tumor expression of HLA Class I and non-classical HLA-E and HLA-G. All markers were, separate and combined, analyzed for clinical prognostic value.

**Results:**

Expression of HLA class I (DFS HR 0.637 (0.458-0.886), p = 0.013), Foxp3+ infiltration above median (OS HR 0.637 (0.500-0.813), p < 0.001 and DFS HR 0.624 (0.491-0.793), p < 0.001) and expression of HLA-G (DFS HR 0.753 (0.574-0.989), p = 0.042) were related to a better clinical prognosis. When these markers were combined, patients with 2 or 3 markers associated with poor prognosis (loss of HLA Class I, Foxp3+ below median, and weak HLA-G expression), showed a significantly worse survival (OS and DFS p < 0.001). This immune-phenotype was an independent predictor for DFS (HR 1.56 (1.14-2.14), p = 0.019).

**Conclusions:**

In conclusion, rectal tumors showing loss of HLA class I expression, Foxp3+ infiltration below median and weak HLA-G expression were related to a worse OS and DFS. Combining these immune markers lead to the creation of tumor immune-phenotypes , which related to patient outcome and were significant independent clinical prognostic markers in rectal cancer.

## Background

The immune system has proven to play an important role in tumorigenesis and gained a lot of attention in cancer research [1-4]. Consequently, evasion of immune surveillance has become one of the important hallmarks of cancer [5]. Tumors are thought to be ‘edited’ through a Darwinian selection process into poorly immunogenic tumor variants, invisible to the immune system and able to grow progressively. Immuno-editing might influence patient’s prognosis substantially [6].

We have described a few mechanisms responsible for evasion of immune surveillance below.

First, cytotoxic T-cells (CTL) are capable of destroying tumor cells by recognizing tumor-associated antigens (TAA) on the tumor cell surface presented by classical human leukocyte antigen (HLA) class I. Tumor cells can escape this CTL recognition through downregulation or complete loss of HLA class I, resulting in minimization of TAA expression and absence of CTL destruction [7-9]. Second, non-classical HLA-E and HLA-G also play an important role in immune surveillance. Presence of HLA-E and HLA-G causes an inhibitory signal to natural killer (NK) cells, resulting in further immune escape [7,10-14]. HLA-E is regularly expressed in various healthy tissues and correlates with HLA class I expression [15]. HLA-G is rarely found in healthy tissues, but is frequently observed in tumors [16]. Third, immune reactivity can become suppressed by the attraction of immunosuppressive regulatory T cells (Tregs) into the tumor microenvironment [17,18].

In colorectal cancer (CRC), the presence of Tregs in the tumor micro-environment has been related to a worse prognosis in some studies, although other studies showed an inverse association [19-22]. Loss of HLA Class I tumor expression was related to a better prognosis in CRC in most studies [14,23] and HLA-E and HLA-G tumor expression has been correlated with a poor prognosis and tumor progression [24,25].

In rectal cancer specifically, only a few studies reported on the role of the immune system, in which expression of HLA Class I was related to a better prognosis [26,27]. Recently, more studies showed differences in biology between colon- and rectal cancer [28-30]. Unfortunately, most studies so far have focused on CRC and did not perform separate analyses. Furthermore, often only one immune marker was investigated in CRC, while recent studies showed the complex interaction between the different mechanisms of immune-escape [6,31,32].

In this study we therefore aimed to investigate the immune-related biomarkers HLA Class I, HLA-E and -G and Tregs, determined with immunohistochemistry, in rectal cancer specifically, and to establish distinct patterns that reflect immune-escape mechanisms of rectal cancer by combining these markers and relate these patterns to clinical outcome.

## Methods

### Study population

The study cohort consisted of patients obtained from the non-preoperative treated arm of the Dutch TME trial (January 12^th^, 1996, DUT-KWF-CKVO-9504, EORTC-40971, EU-96020), a multicenter trial that evaluated total mesorectal excision (TME) surgery with or without preoperative radiotherapy (5 × 5 Gray) from 1996–1999 [33]. Radiotherapeutical, surgical and pathological procedures were standardized and quality-controlled [34]. Before the start of the TME trial the Medical Ethical Committee of the Leiden University Medical Center approved the trial and retrospective use of samples. Written informed consent for participation and retrospective use of samples was obtained from all patients enrolled in the TME trial. Previously, a tissue microarray (TMA) including 1208 patients (irradiated and non-irradiated) of the Dutch TME trial was available. Because of insufficient tissue on this TMA a new TMA was constructed for this study. Sufficient formalin-fixed paraffin-embedded tumor material was available for 495 non-preoperative radiotherapy-treated stage I-IV Dutch patients, resulting in a total study cohort of 495 rectal cancer patients who only had surgery.

### Antibodies

The mouse monoclonal antibodies HCA2 and HC10 were used, which recognize the heavy chains of HLA Class I, these were kindly provided by Prof. Dr. J. Neefjes (NKI, Amsterdam, The Netherlands). The reactivity spectrum of HCA2 comprises all HLA-A chains (except HLA-A24), as well as some HLA-B, HLA-C, HLA-E, HLA-F, and HLA-G chains. HC10 reacts with HLA-B and HLA-C heavy chains and some HLA-A chains (HLA-A10, HLA-A28, HLA-A29, HLA-A30, HLA-A31, HLA-A32, HLA-A33) [31]. The mouse antibody against human Foxp3 (ab20034 clone 236A/E7; Abcam) was used for Treg identification. The reactivity spectrum of Foxp3 is composed of regulatory T cells and may include small numbers of CD8+ cells but is generally considered to be the best single marker for Treg identification [35,36]. For HLA-E and HLA-G identification mouse monoclonal antibodies against HLA-E (ab2216 clone MEM-E/02: AbCam) and HLA-G (4H84: Exbio, Czech Republic) were used [32]. MEM-E/02 recognizes denatured HLA-E [37,38], while 4H84 recognizes denatured HLA-G molecules and also binds to free heavy chains of classical HLA class I molecules [38-40].

### TMA production and immunohistochemistry

Histo-pathological characteristics of tumor material from all patients were standardized and quality-controlled [33,34]. Sections from formalin-fixed paraffin-embedded (FFPE) tumor blocks of the primary tumors were cut for haematoxylin and eosin staining. Based on these slides, histopathologically representative tumor regions were identified and punched for preparation of tumor tissue microarray (TMA) blocks. From each donor block, three 1.0 mm diameter tissue cores were punched from three different identified tumor areas to account for tumor heterogeneity and transferred into a receiver paraffin block using the TMA master (3DHISTECH, Budapest, Hungary). Immunohistochemical staining (IHC) for Foxp3+ cells, non-classical HLA-E and HLA-G, and classical HLA class I tumor expression was performed on 4 μm sections, which were cut from each receiver block and mounted on glass. For each type of primary antibodies, all slides were stained simultaneously to avoid inter-assay variation.

The sections were deparaffinized and rehydrated in accordance with standard protocol. Endogenous peroxidase was blocked for 20 minutes in 0.3% hydrogen peroxide in PBS. For antigen retrieval, slides for staining with HLA-E, HLA-G or Foxp3+ were boiled in a 0.01 M EDTA buffer (pH 8) for 10 minutes at maximum power in a microwave oven. Slides for staining with HCA2 and HC10 were boiled in a 0.1 M citrate buffer (pH 6). Sections were incubated overnight with Foxp3, HLA-E, or HLA-G antibodies at pre-determined optimal dilution. The next day, after 30 minutes of incubation with Envision anti-mouse (K4001; DAKO Cytomation, Glostrup, Denmark), sections were visualized using diaminobenzidine solution (DAB). Tissue sections were counterstained with haematoxylin, dehydrated and finally mounted in pertex.

For the HCA2 and HC10 stainings a double staining was performed to better discriminate between stroma (using a mixture of anti-extracellular matrix antibodies that resulted in brown staining of tumor stroma) and tumor tissue (using a blue staining for the HLA expression to be determined) in the tissue sections. Sections were incubated overnight at room temperature with all primary antibodies simultaneously (anti-collagen I, anti- collagen VI, anti-elastin (all polyclonal rabbit antibodies obtained from AbCam) and HCA2 and HC10). Afterwards, sections were washed three times for 5 minutes in PBS and incubated for 30 minutes with Envision + System HRP anti Rabbit (DAKO, Glostrup, Denmark). After washing the sections three times with PBS, sections were developed using Liquid DAB + Substrate Chromogen System (DAKO, Glostrup, Denmark) following manufacturer’s instructions for visualization of stromal tissue. Then, sections were washed again three times for 5 minutes in PBS followed by 30 minutes incubation with rabbit-anti-mouse antibodies (DAKO, Glostrup, Denmark). Afterwards, the sections were incubated with APAAP (DAKO, Glostrup, Denmark) diluted in PBS/BSA 1% for 30 minutes. And finally, sections were washed three times for 5 minutes in PBS followed by 20 minutes incubation with Vector-Blue following manufacturer’s instructions for visualization of the HCA2 and HC10 antibodies, and mounted in Aquamount (Merck, Darmstadt, Germany).

For each patient, normal epithelium, stromal cells, or lymphoid cells served as internal positive control for HLA class I and HLA-E antibody reactivity [24]. Tonsil tissue served as external positive control for the HCA2 and HC10 stainings and placenta tissue slides for the HLA-E and HLA-G stainings. Slides from human tonsil tissue served as positive control for Foxp3 staining. Tissue slides that underwent the whole immuno-histochemical staining without primary antibodies served as negative controls (Additional file 1: Figure S1).

### Evaluation of immunohistochemistry

Microscopic analysis of HCA2, HC10, HLA-E and HLA-G expression and presence of Foxp3+ cells was performed by two independent observers in a blinded manner (M.S.R.: 100% of the cohort, C.C.E. 30% of the cohort). The kappa values for inter-observer agreement were all between 0.5 and 0.7, indicating substantial agreement between the two observers [41]. The scores of the three 1.0 mm punches were averaged. For HCA2 and HC10 the percentage of tumor cells with membranous staining was assessed. HLA class I expression status was determined according to the International HLA and Immunogenetics Workshop [42], with tumor cell HLA class I expression status defined as follows: loss of HLA class I expression: less than 5% of tumor cells expressing both HCA2 and HC10, downregulation of HLA class I; less than 5% of tumor cells expressing either of the markers, and expression of HLA class I: 5% or more expressing both markers. For HLA-E and HLA-G, intensity of tumor staining (absent (undetectable or faint in <20% of the cells), weak (faint to weak in 20% but ≤70% of the cells), moderate (weak to moderate in >70% of the cells) or strong intensity (intense in 20-70% of the cells)) was determined, based on previous studies [43,44]. The scores of the three 1.0 mm punches were averaged as well. For analysis these scores were further categorized as weak (absent and weak intensity together) versus strong (moderate and strong intensity together) tumor staining. Quantification of the number of Foxp3+ cells was microscopically assessed in the entire tumor punches of the TMA and the absolute number of positive cells was used for analysis, with the use of the median as cut-off value for categorization in two categories: Foxp3+ below median and Foxp3+ above median.

### Statistical analysis

Statistical analyses were performed using the statistical package SPSS (version 17.0 for Windows; SPSS Inc.). The Student’s T-test and the Chi-squared test were used to evaluate associations between tumor expression of classical HLA Class I, non-classical HLA-E and HLA-G and tumor infiltration of Foxp3+ cells and various clinico-pathological variables. Overall Survival (OS) was defined as time of surgery until death; Disease Free Survival (DFS) as time of surgery until death or relapse of disease, whichever came first. The Kaplan-Meier method was used for calculation of survival probabilities and the Log-rank test for comparison of survival curves between expression levels of markers. Cox regression was used for univariate and multivariable analysis for OS and DFS. To preserve statistical power in subgroup analyses, patients with stage IV disease (n = 32) and positive resection margin (n = 98) were included in the final analyses. In multivariable analyses corrections were made for TNM stage, circumferential margin, age, tumor grade and adjuvant therapy.

## Results

### HLA class I tumor expression

**Figure 1:**
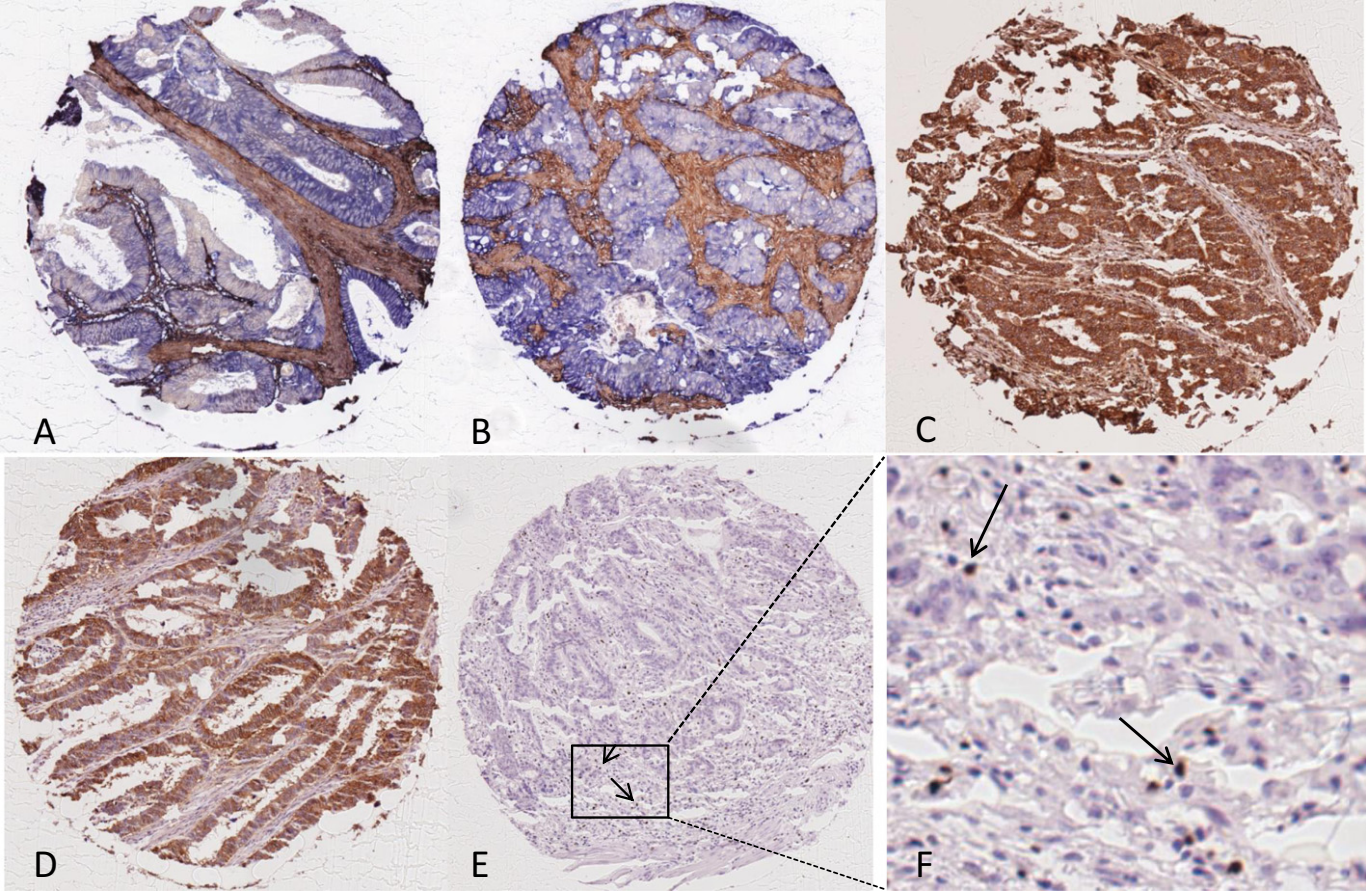
**Representative images of HCA2, HC10, HLA-E and –G and Foxp3+ staining in rectal cancer.** Representative images of immunohistochemical stainings for HLA Class I expression (HCA2 and HC10), HLA-E and HLA-G expression and presence of Foxp3+ cells, performed according to standard protocols (details in Material and Methods). **(A)** HCA2-positive tumor (note: positive tumor cells in blue, stromal cells are stained brown); **(B)** HC10-positive tumor (note: positive tumor cells in blue, stromal cells are stained brown); **(C)** HLA-E positive tumor (note: positive tumor cells in brown); **(D)** HLA-G positive tumor (note: positive tumor cells in brown); **(E)** Presence of Foxp3+ cells (two representative examples of Foxp3+ cells are indicated by arrows) with a magnification in **(F)**.

**Figure 2:**
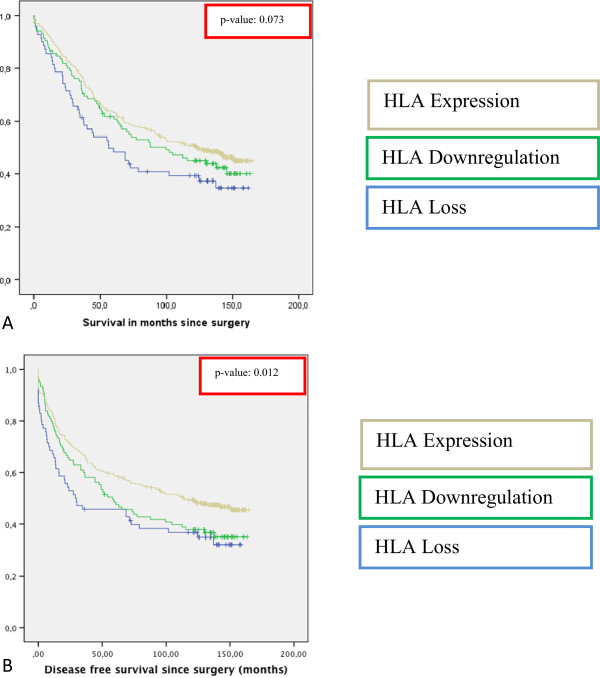
**Survival curves stratified for HLA class I tumor expression in rectal cancer. A)** Kaplan Meier curve for Overall Survival in 495 rectal cancer patients stratified for HLA class I tumor expression status. **B)** Kaplan Meier curve for Disease Free Survival in 495 rectal cancer patients stratified for HLA Class I tumor expression. HLA class I was immunohistochemically determined as described in the Material and Methods section.

The analysis of HLA class I expression was performed on 468 stage I-IV rectal cancer patients as, due to staining artifacts and loss of material during the staining procedure, the IHC results of 27 cases could not be analyzed. Representative images of HLA Class I expression are shown in Figure 1. Loss of HLA Class I expression was seen in 70 patients out of 468 patients (15%), down regulation in 105 patients (22%) and expression was present in the majority of the cases: 293 patients (63%). Patient characteristics and data on HLA class I expression are shown in Table 1. Patients with loss of HLA class I tumor expression were diagnosed significantly more often with stage IV tumors (p = 0.001) and T3 or T4 tumors (p = 0.016). Also, loss of HLA class I was related to more nodal involvement (p = 0.003), tumors with poor differentiation (p = 0.033) and more adjuvant treatment (p = 0.001).HLA class I expression was borderline significantly related to a better OS (logrank p-value 0.073), but also significantly related to a better DFS (logrank p-value 0.012) with a HR of 0.637 (95% CI 0.458-0.886, p = 0.013) for expression of HLA class I compared to loss of HLA class I expression (Figure 2).

**Table 1:** Patient Characteristics of the Total Rectal Cancer Cohort and stratified for HLA class I, HLA-G and Foxp3+ expression

	**Total population n = 495**	** HLA Class I Loss n = 70 (15%)**	**HLA Class I Downregulationn = 105 (22%)**	**HLA Class I Expression n = 293 (63%)**	**HLA-G Weak n = 350 (72%)**	**HLA-G Strong n = 134 (28%)**	**Foxp3+ Below median n = 240 (50%)**	**Foxp3+ Above median n = 238 (50%)**
**Gender (%)**								
**Male**	316 (63.8%)	49 (70.0%)	63 (60.0%)	186 (63.5%)	227 (64.9%)	83 (61.9%)	162 (67.5%)	142 (59.7%)
**Female**	179 (36.2%)	21 (30%)	42 (40.0%)	107 (36.5%)	123 (35.1%)	51 (38.1%)	78 (32.5%)	96 (40.3%)
**Age in years (mean SD)**	64.5 (11.3)	64.8 (12.2)	65.5 (11.0)	64.0 (11.0)	64.7 (11.1)	63.9 (11.7)	64.7 (11.9)	(64.2 (10.5)
**TNM stage (%)**								
**I**	134 (27.1%)	9 (12.9%)	27 (25.7%)	89 (30.4%)	80 (22.9%)	50 (37.3%)	43 (17.9%)	85 (35.7%)
**II**	136 (27.5%)	19 (27.1%)	25 (23.8%)	90 (30.7%)	98 (28.0%)	37 (27.6%)	65 (27.1%)	71 (29.8%)
**III**	193 (39.0%)	33 (47.1%)	51 (48.6%)	96 (32.8%)	146 (41.7%)	41 (30.6%)	112 (46.7%)	72 (30.3%)
**IV**	32 (6.5%)	9 (12.9%)	2 (1.9%)	18 (6.1%)	26 (7.4%)	6 (4.5%)	20 (8.3%)	10 (4.2%)
**Tumor grade (%)**								
**Moderate**	358 (72.3%)	41 (58.6%)	72 (68.9%)	228 (77.8%)	248 (70.9%)	102(76.1%)	160 (66.7%)	185 (77.7%)
**Poor**	110 (22.2%)	23 (32.9%)	25 (23.8%)	54 (18.4%)	83 (23.7%)	25 (18.7%)	67 (27.9%)	40 (16.8%)
**Well**	25 (5.1%)	5 (7.1%)	8 (7.6%)	10 (3.4%)	17 (4.9%)	7 (5.2%)	12 (5.0%)	12 (5.0%)
**Missing**	2 (0.4%)	1 (1.4%)		1 (0.3%)	2 (0.6%)		1 (0.4%)	1 (0.4%)
**Adjuvant therapy**								
**No**	402 (81.2%)	46 (65.7%)	84 (80%)	253 (86.3%)	278 (79.4%)	116 (86.6%)	185 (77.1%)	205 (86.1%)
**Yes**	75 (15.2%)	19 (27.1%)	19 (18.1%)	31 (10.6%)	58 (16.6%)	14 (10.4%)	44 (18.3%)	28 (11.8%)
**Missing**	18 (3.6%)	5 (7.1%)	2 (1.9%)	9 (3.1%)	14 (4.0%)	4 (3.0%)	11 (4.6%)	5 (2.1%)
**Circumferential margin**								
**Negative**	397 (80.2%)	49 (70.0%)	83 (79.0%)	242 (82.6%)	279 (79.7%)	110 (82.1%)	188 (78.3%)	196 (82.4%)
**Positive**	98 (19.8%)	21 (30.0%)	22 (21.0%)	51 (17.4%)	71 (20.3%)	24 (17.9%)	52 (21.7%)	42 (17.6%)

This table shows the patient characteristics of the entire rectal cancer cohort (n = 495) and stratified for HLA class I, HLA-G and Foxp3+ staining.

### Tumor infiltrating Foxp3+ cells

**Figure 3:**
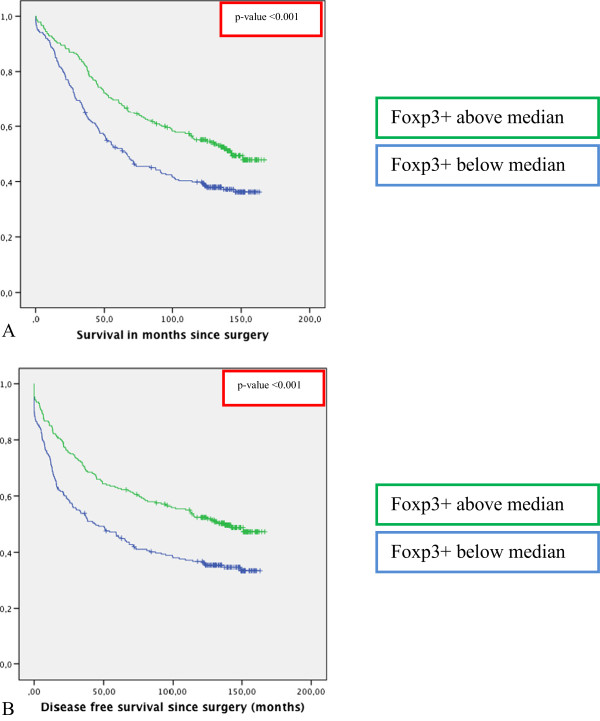
**Survival curves stratified for Foxp3+ tumor infiltration in rectal cancer. A)** Kaplan Meier curve for Overall Survival in 495 rectal cancer patients stratified for Foxp3+ tumor infiltration based on the median of the total Foxp3+ infiltration in this cohort. **B)** Kaplan Meier curve for Disease Free Survival in 495 rectal cancer patients stratified for Foxp3+ tumor infiltration. Foxp3+ tumor infiltration was immunohistochemically determined as described in the Material and Methods section.

The number of Foxp3+ cells was evaluated in 478 patients, as, due to staining artifacts and loss of material during the staining procedure, the IHC results of 17 cases could not be analyzed. Representative images of Foxp3 staining are shown in Figure 1 and patient characteristics and data on Foxp3+ tumor infiltration are shown in Table 1. The mean number Foxp3+ cells per tumor punch was 39 with a median of 27.0. For further analysis Foxp3+ was categorized as below vs. above median due to skewness in the spread of the data. This resulted in 240 patients with presence of Foxp3+ cells below median and 238 patients with presence of Foxp3+ cells above median. Tumors with Foxp3+ cells above median were significantly more often stage I tumors (p < 0.001), T1 or T2 tumors (p < 0.001) and showed less nodal involvement (p = <0.001). Poorly differentiated tumors were associated with tumors with presence of Foxp3+ cells below median (p = 0.022). Furthermore, tumors with expression of HLA class I showed significantly more Foxp3+ cells above median compared to tumors with loss of HLA class I expression (p < 0.001).The presence of Foxp3+ cells above median in the tumor microenvironment was significantly related to a better OS (logrank p-value <0.001) and DFS (logrank p-value <0.001) with HR’s of 0.637 (95% CI 0.500-0.813, p < 0.001) and 0.624 (95% CI 0.491-0.793, p < 0.001) respectively in case of presence of Foxp3+ cells above median compared to Foxp3+ cells below median (Figure 3).

### HLA-E and HLA-G tumor expression

The analysis of HLA-E and HLA-G was performed on 486 and 484 patients respectively, as, due to staining artifacts and loss of material during the staining procedure, the IHC results of 9 and 11 cases respectively, could not be analyzed. Representative images of non-classical HLA-E and HLA-G immunohistochemical staining results are shown in Figure 1. For HLA-E, 8 patients (1.6%) showed absence of tumor staining, 73 patients (15.0%) showed weak tumor staining, 298 patients (61.3%) showed moderate tumor staining and 107 patients (22.0%) showed strong tumor staining in their punches. For HLA-G, 31 patients (6.4%) had absence of tumor staining, 319 patients (65.9%) had a weak tumor staining, 103 patients (21.3%) had a moderate tumor staining and 31 patients (6.4%) had a strong tumor staining. For analysis the scores were further categorized as weak (absent and weak intensity together) versus strong (moderate and strong intensity together) tumor staining. Strong expression was found in 83.3% (405 out of 486) of the tumors for HLA-E and in 27.7% (134 out of 484) of the tumors for HLA-G expression. Weak expression of HLA-E was significantly related to T4 tumors (p = 0.020) and more nodal involvement (p = 0.050). Weak expression of HLA-G was also significantly related to higher tumor stage (p = 0.008) and more nodal involvement (p = 0.006). Furthermore, strong expression of HLA-G was significantly associated with presence of Foxp3+ cells above median (p = 0.001) and with HLA class I expression (p < 0.001). Strong HLA-E was also significantly related to HLA class I expression (p = 0.028).

HLA-E expression was not related to OS (p = 0.823) or DFS (p = 0.784). Strong expression of HLA-G was borderline significantly related to a better OS (logrank p-value 0.056) and significantly related to a better DFS (logrank p-value 0.040) with a HR of 0.753 (95% CI 0.574-0.989, p = 0.042) in case of strong expression of HLA-G compared to weak expression of HLA-G.

### Multivariable analysis

A multivariable analysis was performed for OS and DFS using the following parameters: age, TNM stage, tumor grade, adjuvant therapy, circumferential margin, HLA class I expression status, HLA-G expression status and Foxp3+ tumor infiltration. Foxp3+ was an independent significant predictor of OS (p = 0.018) and DFS (p = 0.012). HLA Class I and HLA-G were not significantly related to OS and DFS in multivariable analysis. In Table 2 all univariate and multivariable analyses are summarized.

**Table 2:** Univariate and multivariable analyses of Disease Free Survival (DFS) and Overall Survival (OS) for the different immune markers and for tumor immune phenotypes

**DFS**							**OS**					
**Univariate analysis**				Multivariable analysis**			**Univariate analysis**			Multivariable analysis**		
	**HR**	**95% CI**	**p-value**	**HR**	**95% CI**	**p-value**	**HR**	**95% CI**	**p-value**	**HR**	**95% CI**	**p-value**
HLA class I			0.013*			0.548			0.075			0.984
Loss	1.00			1.00			1.00			1.00		
Downregulation	0.84	0.58-1.22		1.08	0.72-1.62		0.78	0.53-1.15		1.04	0.68-1.59	
Expression	0.64	0.46-0.89		0.91	0.63-1.32		0.68	0.49-0.95		1.03	0.70-1.52	
Foxp3+			<0.001*			0.012*			<0.001*			0.018*
Below median	1.00			1.00			1.00			1.00		
Above median	0.62	0.49-0.79		0.72	0.56-0.93		0.64	0.50-0.81		0.73	0.56-0.95	
HLA-G			0.042*			0.849			0.056			0.418
Weak expression	1.00			1.00			1.00			1.00		
Strong expression	0.75	0.57-0.99		0.85	0.63-1.13		0.76	0.58-1.01		0.88	0.66-1.19	
Immune phenotype			<0.001*			0.019*			<0.001*			0.122
Phenotype 1	1.00			1.00			1.00			1.00		
Phenotype 2	1.26	0.94-1.68		1.13	0.83-1.54		1.18	0.87-1.58		1.07	0.78-1.47	
Phenotype 3	2.06	1.54-2.75		1.56	1.14-2.14		1.88	1.40-2.53		1.39	1.01-1.92	
TNM stage			<0.001*						<0.001*			
I-II	1.00						1.00					
III-IV	2.65	2.09-3.37					2.65	2.08-3.38				
Circumferential margin			<0.001*						<0.001*			
Negative	1.00						1.00					
Positive	2.26	1.73-2.94					2.42	1.85-3.16				
Age (continuous)	1.03	1.02-1.04	<0.001*				1.04	1.03-1.05	<0.001*			
Tumor grade			0.094						0.058			
Well	1.00						1.00					
Moderate	0.88	0.53-1.46					0.87	0.52-1.44				
Poor	1.19	0.69-2.04					1.21	0.70-2.09				
Adjuvant chemotherapy			<0.001*						<0.001*			
No	1.00						1.00					
Yes	2.38	1.77-3.20					2.43	1.80-3.28				

*Statistical significance.

**Corrected for TNM stage, circumferential margin, age, tumor grade and adjuvant therapy.

Because the type of antibody we used to detect HLA-G expression is known to bind to free heavy chains of classical HLA class I molecules as well (38–40), interaction between these two markers was analysed for survival. In multivariable analysis for OS there was no interaction between HLA-G expression and HLA class I expression (p = 0.174). Also, there was no interaction between HLA-G expression and the two types of antibodies used for detection of HLA class I separately; HCA2 expression (p = 0.183) and HC10 expression (p = 0.461) respectively. For DFS, there was no interaction between HLA-G expression and HLA class I expression as well (p = 0.301), neither for HCA2 (p = 0.516) nor HC10 (p = 0.329).

### Analysis of tumor immune-phenotypes

**Figure 4:**
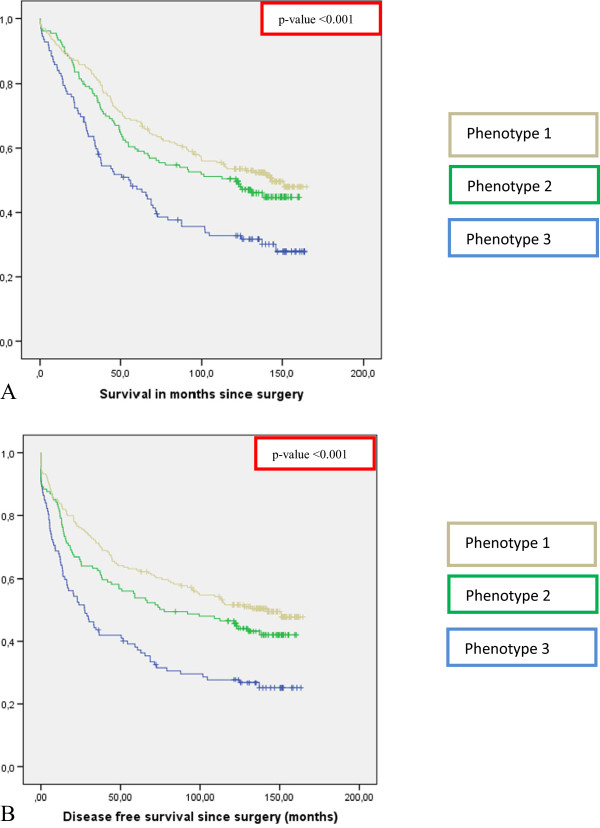
**Survival curves stratified for immune-phenotypes in rectal cancer. A)** Kaplan Meier curve for Overall Survival in 495 rectal cancer patients stratified for all the different combinations between tumor expression of HLA class I, HLA-G and the presence of Foxp3+ cells based on which 3 immune-phenotypes could be distinguished. See results section for explanation of the phenotypes. **B)** Kaplan Meier curve for Disease Free Survival in 495 rectal cancer patients stratified for all the different combinations between tumor expression of HLA class I, HLA-G and the presence of Foxp3+ cells based on which 3 immune phenotypes could be distinguished. See results section for explanation of the phenotypes.

The interaction between tumor cells and immune cells is complex, multifaceted and different interactions are closely linked to each other. In breast- and colon cancer patients, immune subtyping has already shown a promising value in the prediction of prognosis [44,45]. Therefore, we hypothesized that combined analysis of immune markers may better reflect patients’ outcome as a result of interaction between tumor cells and the immune system in rectal cancer as well. We have shown above that patients with tumors showing expression of HLA class I, expression of HLA-G and presence of Foxp3+ cell infiltration above median showed better survival outcomes when analyzed separately. HLA-E tumor expression was not related to survival. Based on the prognostic value of the individual markers, a score was created for the combination of HLA class I, HLA-G and Foxp3+. HLA class I was divided into 3 scores, which ranged from 0 for loss of expression to 2 for high expression. HLA-G and Foxp3+ were divided into 2 scores; 0 for weak HLA-G expression or Foxp3+ below median and 1 for strong HLA-G expression or Foxp3+ above median. Combining the scores of the individual markers resulted in a scoring range from 0 to 4. The entire population was divided into 3 tumor immune-phenotypes: patients with scores 3 and 4 (phenotype 1, n = 210), patients with score 2 (phenotype 2, n = 139) and patients with scores 0 and 1 (phenotype 3, n = 112).In survival analyses, these phenotypes showed significant differences in patient outcome. Survival outcome increased with an increasing number of positive prognostic immune markers expressed in the tumor. Patients with phenotype 3 showed a significantly worse OS (logrank p < 0.001) and DFS (logrank p < 0.001) with HR’s of 1.88 (95% CI 1.40-2.53, p < 0.001) for OS and 2.06 (95% CI 1.54-2.75, p < 0.001) for DFS, when compared to phenotype 1 (Figure 4).

### Multivariable analysis of the tumor immune-phenotypes

For the tumor immune-phenotype, univariate analysis and multivariable analysis was also performed to determine OS and DFS as written above. In univariate analysis the immune-phenotype was a significant predictor of OS (p < 0.001) and DFS (p < 0.001) (Table 2). In multivariable analysis the immune-phenotype was an independent predictor of DFS (p = 0.019). It was not an independent predictor of OS (p = 0.122). When compared to the multivariable analyses of the individual immune markers as shown in Table 2, the combination between immune markers, the tumor immune-phenotype, showed a stronger and additive prognostic potential, indicating a complex and multifaceted interaction between tumor cells and immune cells.

## Discussion

In this study, by combining the immune-related tumor markers HLA class I, HLA-G and Foxp3+, we reported an independent association between tumor immune-phenotype and patient outcome. These phenotypes might represent how the immune system controls tumor growth and metastases in rectal cancer.

Previous studies on HLA class I expression, which focused on a mixed population of colon- and rectal cancer together, have shown inconsistent findings [13,14]. Our study showed a survival benefit for patients with tumors expressing HLA class I. These results are partly comparable with results from Watson *et al*., who showed that low expression of HLA class I was related to a poor prognosis in a large group of colorectal cancer patients, whereas tumors with loss or expression of HLA class I were associated with a survival benefit [14]. A substantial part of Watson’s cohort showed HLA class I negative tumors (24.6%). In our cohort, consisting solely of rectal cancer patients, only 15.0% of the patients had tumors with loss of HLA class I expression, which might indicate that colon cancers lose their HLA class I expression more often. Previously, Speetjens *et al.* investigated the prognostic value of HLA class I expression in rectal cancer patients from the Dutch TME Trial as well [27]. In this study, as described in the methods sections, a new TMA was used without complete overlap and thus different patients. Both studies showed a survival benefit for patients with tumors showing expression of HLA class I. Because we have changed the scoring criteria based on recommendation by the International HLA and Immunogenetics Workshop [42] differences have to be acknowledged. Speetjens *et al.* reported that 16% of non-irradiated patients had tumors with loss and downregulation of HLA Class I, whereas our study showed 37% (15% loss and 22% downregulation). Thus, besides a different patient cohort, other possible explanations for inconsistent findings between studies are the use of different definitions of HLA class I expression and differences in staining techniques. Furthermore, tumor microsatellite status might also play an important role. Approximately 50% of all proximal colon tumors show microsatellite instability (MSI), whereas almost all distal colon and rectal cancers are microsatellite stable (MSS) tumors [46,47]. Loss of HLA class I has been described more significantly in MSI colorectal tumors compared to MSS right-sided colon tumors [48,49]. HLA class I negative tumors are therefore more likely to be MSI tumors with a different clinical behavior than MSS colorectal tumors [27]. Since MSI tumors have a better prognosis, MSI might influence prognostic results when considering HLA class I expression in colorectal tumors [46]. In this rectal cancer cohort determination of the microsatellite status would not have been useful. Research has shown that in only 2% of rectal cancers MSI can be found [50], resulting in insufficient statistical power for separate analyses Finally, colon and rectum are biologically different tissues; the colon epithelium consists of simple columnar epithelium, whereas the rectum is a transition from single columnar epithelium to stratified squamous epithelium, which might result in different outcomes. The Cancer Genome Atlas Network attempted to find biological differences between colon and rectal cancer. However, only differences in anatomical tumor site with more hypermethylation in right-sided tumors were found, which might be explained by different embryonic origins of the right-and left-sided tumors [28].

Results in our study regarding non-classical HLA-G are remarkable. HLA-G expression can inhibit NK-cells from lysing tumor cells that have lost or downregulated classical HLA class I expression as a secondary immune escape [51,52]. However, in this study, positive HLA-G expression was correlated with a longer disease free survival.

The antibody used to stain HLA-G can also bind to free heavy chains of classical HLA class I molecules as well, possibly explaining the remarkable results. We therefore performed an interaction analysis between these antibodies. However, no interaction between HLA-G and HLA class I expression was found. Furthermore, HLA-G is found to be highly immunosuppressive by directly inhibiting NK cells, but also by recruitment of Tregs and induction of Treg differentiation [53]. Our study showed that strong HLA-G expression was significantly related to presence of more Foxp3+ cells, possibly explaining the favourable prognosis of tumors with strong HLA-G expression, since tumors that attracted more Foxp3+ cells had a better outcome in our cohort. Immune regulation in cancer still remains complex and multifaceted, and not all immune related mechanisms are completely clear. To our knowledge, no other studies on HLA-E and HLA-G are performed on rectal cancer specifically and therefore no other comparisons could be made.

The presence of Foxp3+ cells in the tumor microenvironment is thought to inhibit host-protective antitumor responses and especially CTL activity [6]. A high density of tumor infiltrating Foxp3+ cells has shown to be associated with an unfavorable prognosis in a wide range of human carcinomas [54,55]. However, in accordance with our results, opposite results are described in CRC [20,21]. A possible explanation could be a significant association between HLA class I tumor expression and Foxp3+ tumor infiltration in our cohort. Foxp3+ infiltrating cells might be necessary to counteract CTL activity in tumors expressing HLA class I to prevent an auto-immune response on other bodily cells as well. Another explanation might be a different micro-environment of rectal cancer, which is colonized with many gastro-intestinal bacteria, triggering the production of pro-inflammatory cytokines causing tumor-enhancing effects. Instead of the specificity of infiltrating T-cells for tumor-antigens, T-cells in rectal cancer could be more specific for the microflora and suppress inflammation and immune responses from bacterial invasion, resulting in an anti-tumorigenic effect [56].

As shown in our results and results from our previous studies in breast cancer, immune markers are related to each other [31,32]. Studying solely one marker might not be enough to truly understand cancer immune surveillance. When we combined our markers, patients showing the worst prognosis were patients with tumors bearing 2 or 3 negative prognostic markers; patients with loss of HLA class I tumor expression, weak HLA-G tumor expression and low tumor infiltration with Foxp3+ cells. These patients therefore qualify as very low immune susceptible. They probably were able to elicit only a minimal CTL attack and subsequently attracted little to no Foxp3+ cells in their tumor micro-environment, possibly explaining their worse prognosis. Furthermore, patients with tumors showing loss of HLA class I expression, low Foxp3+ cell infiltration and strong HLA-G expression showed the worst outcome perspectives. These patients probably had tumors which were highly ‘edited’ as well, causing a minimal CTL attack and subsequently attracted little to no Foxp3+ cells, and because of strong HLA-G expression were able to escape further immune recognition through inhibition of NK cell recognition and subsequently no elimination [51,52].

## Conclusions

In conclusion, we were able to identify local immune escape mechanisms of rectal cancer, where the presence of Foxp3+ infiltration greatly influences a better prognosis. Loss of HLA class I expression, weak non-classical HLA-G expression and the presence of Foxp3+ below median were related to a worse outcome. Combining these immune-related markers identified 3 groups, which were highly selective and discriminative regarding patient outcome. Prognosis increased with a decrease in negative prognostic markers. In the future these findings might contribute to better treatment allocation.

## Competing interests

The authors declare that they have no competing interests.

## Authors’ contributions

MSR participated in the design of the study, carried out the immunohistochemistry and scoring, performed statistical analysis and drafted the manuscript. CCE performed scoring of the immunohistochemical stainings, interpreted the data and aided in drafting the manuscript. HP participated in the design of the study and performed statistical analysis. HM participated in the design of the study, interpreted data and provided pathological support. GJL interpreted data and aided in drafting the manuscript. CJHC participated in the design of the study (principle investigator Dutch TME trial), and interpreted data. PJKK participated in the design of the study, interpreted data and aided in drafting the manuscript. All authors read and approved the final manuscript.

## Pre-publication history

The pre-publication history for this paper can be accessed here:

http://www.biomedcentral.com/1471-2407/14/486/prepub

## Supplementary Material

Additional file 1: Figure S1 Representative images of HCA2, HC10, HLA-E, HLA-G and Foxp3+ staining in rectal cancer. Representative images of immunohistochemical stainings with positive and negative controls for HLA Class I expression (HCA2 and HC10), HLA-E and HLA-G expression and presence of Foxp3+ cells, performed according to standard protocols (details in Material and Methods section). (A) HCA2 expression, positive tumor (note: positive tumor cells in blue, stromal cells are stained brown) (A1), negative tumor (A2), tonsil which served as positive control (A3), tonsil which underwent the whole immuno-histochemical staining without primary antibody served as negative control (A4); (B) HC10 expression, positive tumor (note: positive tumor cells in blue, stromal cells are stained brown) (B1), negative tumor (B2), tonsil which served as positive control (B3), tonsil which underwent the whole immuno-histochemical staining without primary antibody served as negative control (B4); (C) HLA-E expression, positive tumor (note: positive tumor cells are stained brown) (C1), negative tumor (C2), placenta which served as positive control (C3), placenta which underwent the whole immuno-histochemical staining without primary antibody served as negative control (C4); (D) HLA-G expression, positive tumor (note: positive tumor cells are stained brown) (D1), negative tumor (D2), placenta which served as positive control (D3), placenta which underwent the whole immuno-histochemical staining without primary antibody served as negative control (D4); (E) Presence of Foxp3+ cells, tumor with presence of Foxp3+ cells (indicated by arrows) (E1), tumor with absence of Foxp3+ cells (E2), tonsil which served as positive control for Foxp3+ cells (indicated by arrows) (E3), tonsil which underwent the whole immuno-histochemical staining without primary antibody served as negative control (E4). Click here for file
