# Supplementary material for: Prognostic value of HLA class I, HLA-E, HLA-G and Tregs in rectal cancer: a retrospective cohort study
Source: BMC Cancer. 2014 Jul 5;14:486. doi: 10.1186/1471-2407-14-486 (PMC4094545; doi:10.1186/1471-2407-14-486)
Supplement: Additional file 1: Figure S1 — Representative images of HCA2, HC10, HLA-E, HLA-G and Foxp3+ staining in rectal cancer. Representative images of immunohistochemical stainings with positive and negative controls for HLA Class I expression (HCA2 and HC10), HLA-E and HLA-G expression and presence of Foxp3+ cells, performed according to standard protocols (details in Material and Methods section). (A) HCA2 expression, positive tumor (note: positive tumor cells in blue, stromal cells are stained brown) (A1), negative tumor (A2), tonsil which served as positive control (A3), tonsil which underwent the whole immuno-histochemical staining without primary antibody served as negative control (A4); (B) HC10 expression, positive tumor (note: positive tumor cells in blue, stromal cells are stained brown) (B1), negative tumor (B2), tonsil which served as positive control (B3), tonsil which underwent the whole immuno-histochemical staining without primary antibody served as negative control (B4); (C) HLA-E expression, positive tumor (note: positive tumor cells are stained brown) (C1), negative tumor (C2), placenta which served as positive control (C3), placenta which underwent the whole immuno-histochemical staining without primary antibody served as negative control (C4); (D) HLA-G expression, positive tumor (note: positive tumor cells are stained brown) (D1), negative tumor (D2), placenta which served as positive control (D3), placenta which underwent the whole immuno-histochemical staining without primary antibody served as negative control (D4); (E) Presence of Foxp3+ cells, tumor with presence of Foxp3+ cells (indicated by arrows) (E1), tumor with absence of Foxp3+ cells (E2), tonsil which served as positive control for Foxp3+ cells (indicated by arrows) (E3), tonsil which underwent the whole immuno-histochemical staining without primary antibody served as negative control (E4). [file 1471-2407-14-486-S1.doc]

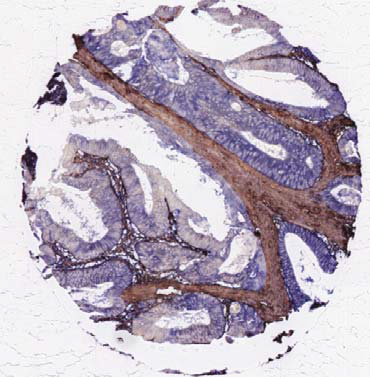

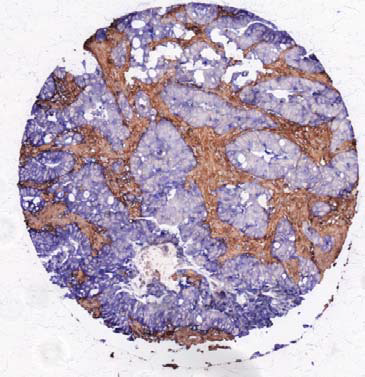

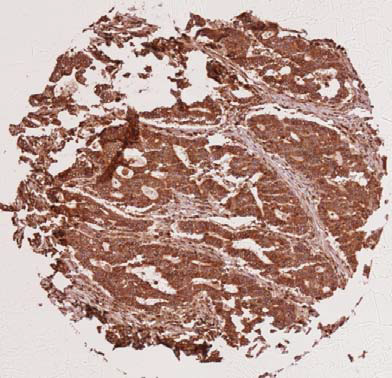

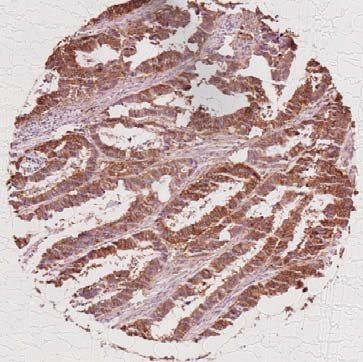

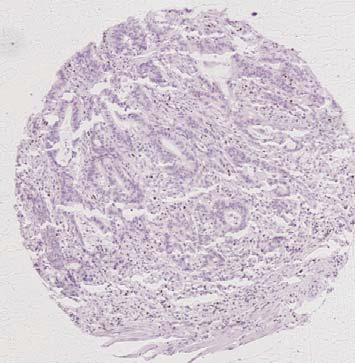

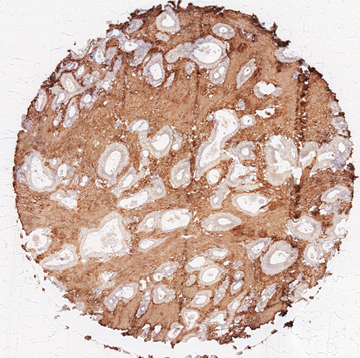

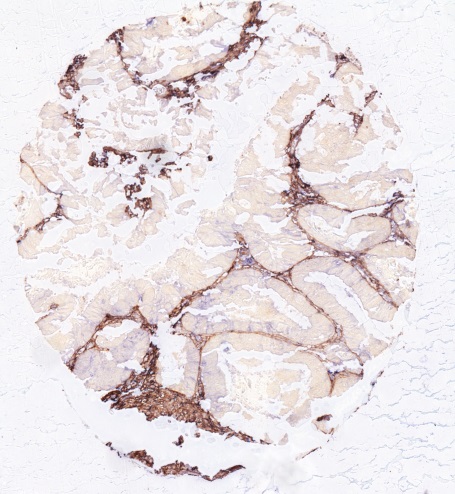

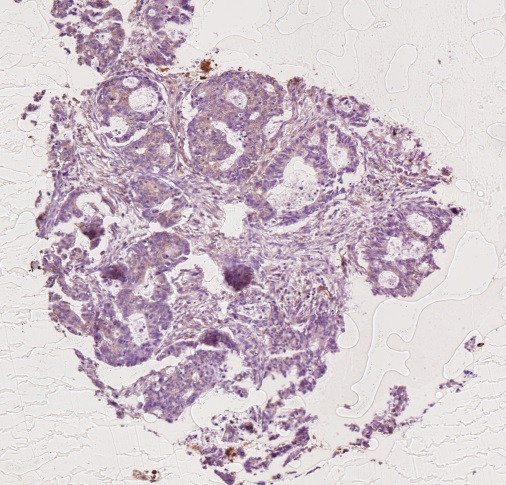

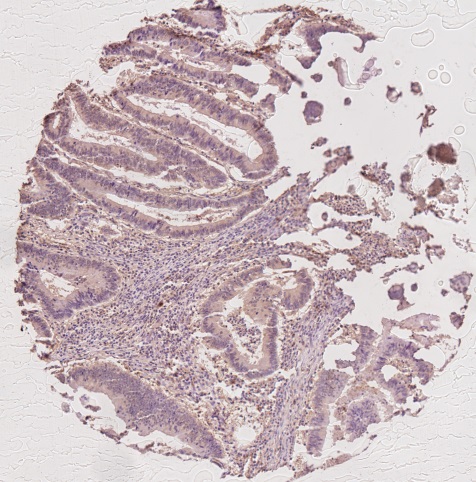

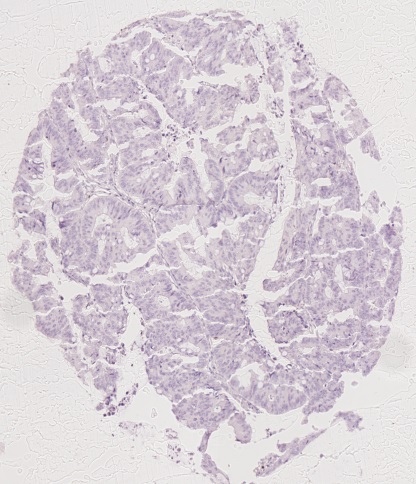

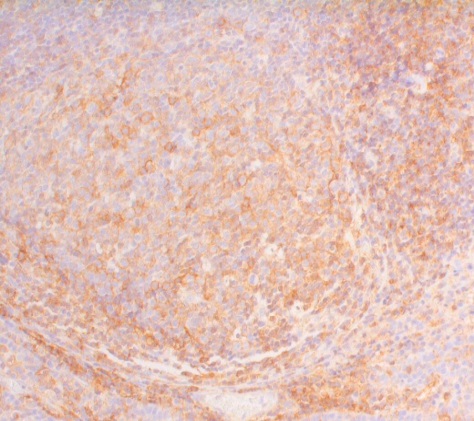

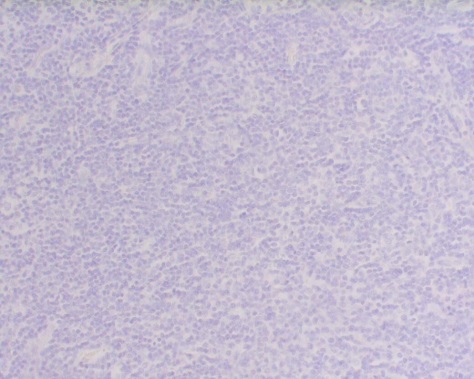

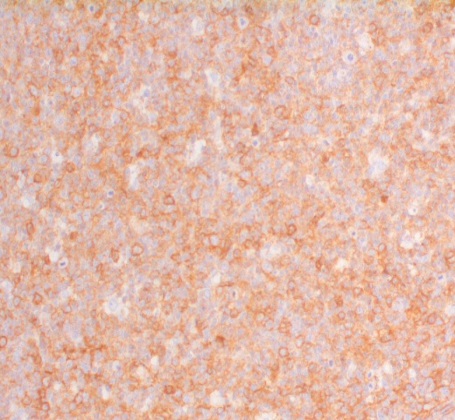

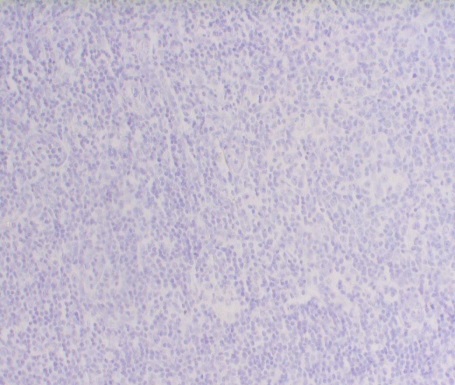

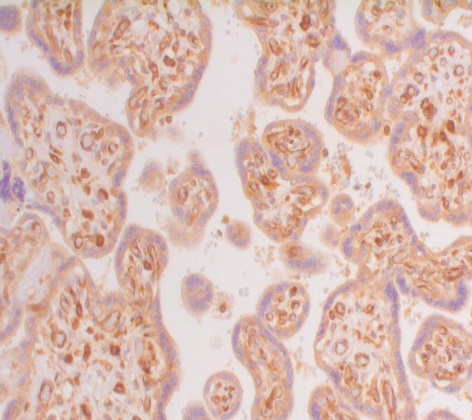

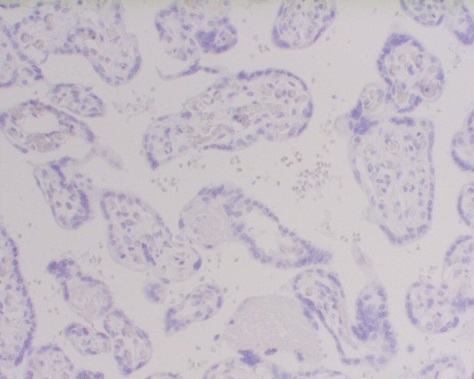

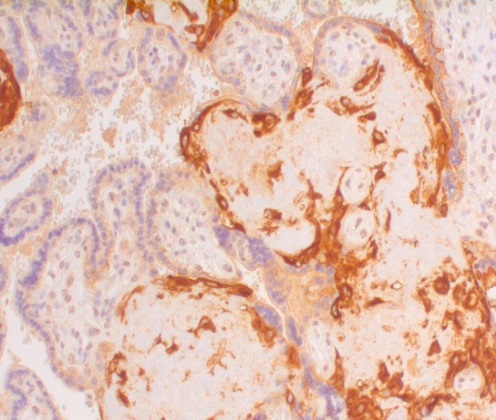

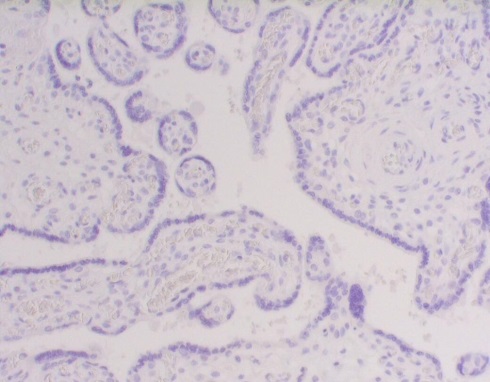

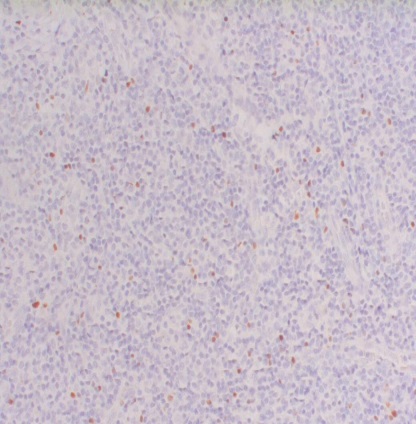

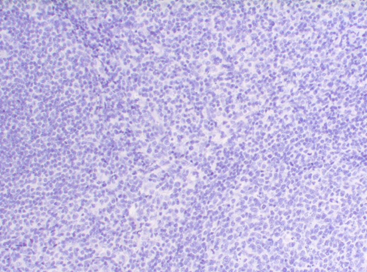


A1

B1

C1

D1

E1

A2

A3

A4

B2

B3

B4

C2

C3

C4

D2

D3

D4

E2

E3

E4

**Supplemental Figure 1**: Representative images of HCA2, HC10, HLA‐E, HLA‐G and Foxp3+ staining in rectal cancer
